# Climate Warming, Marine Protected Areas and the Ocean-Scale Integrity of Coral Reef Ecosystems

**DOI:** 10.1371/journal.pone.0003039

**Published:** 2008-08-27

**Authors:** Nicholas A. J. Graham, Tim R. McClanahan, M. Aaron MacNeil, Shaun K. Wilson, Nicholas V. C. Polunin, Simon Jennings, Pascale Chabanet, Susan Clark, Mark D. Spalding, Yves Letourneur, Lionel Bigot, René Galzin, Marcus C. Öhman, Kajsa C. Garpe, Alasdair J. Edwards, Charles R. C. Sheppard

**Affiliations:** 1 School of Marine Science & Technology, Newcastle University, Newcastle-upon-Tyne, United Kingdom; 2 Wildlife Conservation Society, Marine Programs, Bronx, New York, United States of America; 3 National Research Council, NOAA Panama City Laboratory, Panama City Beach, Florida United States of America; 4 ARC Centre of Excellence for Coral Reef Studies, James Cook University, Townsville, Queensland, Australia; 5 Centre for Environment, Fisheries and Aquaculture Science, Lowestoft Laboratory, Lowestoft, United Kingdom; 6 School of Environmental Sciences, University of East Anglia, Norwich, United Kingdom; 7 Institut de Recherche pour le Développement, Nouméa, New Caledonia; 8 Laboratoire d'Ecologie marine (ECOMAR), Université de La Réunion, Saint-Denis, La Réunion, France; 9 Natural England, Bridgewater House, Whitworth Street, Manchester, United Kingdom; 10 The Nature Conservancy, Newmarket, United Kingdom; 11 Université de la Méditerranée, Centre d'Océanologie de Marseille, UMR CNRS 6540 DIMAR, Campus de Luminy, Case 901, Marseille, France; 12 UMR 5244 CNRS-EPHE-UPVD, Ecosystèmes Coralliens, Université de Perpignan, Perpignan, France; 13 Department of Zoology, Stockholm University, Stockholm, Sweden; 14 School of Biology, Newcastle University, Newcastle-upon-Tyne, United Kingdom; 15 University of Warwick, Department of Biological Sciences, Coventry, United Kingdom; University of Sheffield, United Kingdom

## Abstract

Coral reefs have emerged as one of the ecosystems most vulnerable to climate variation and change. While the contribution of a warming climate to the loss of live coral cover has been well documented across large spatial and temporal scales, the associated effects on fish have not. Here, we respond to recent and repeated calls to assess the importance of local management in conserving coral reefs in the context of global climate change. Such information is important, as coral reef fish assemblages are the most species dense vertebrate communities on earth, contributing critical ecosystem functions and providing crucial ecosystem services to human societies in tropical countries. Our assessment of the impacts of the 1998 mass bleaching event on coral cover, reef structural complexity, and reef associated fishes spans 7 countries, 66 sites and 26 degrees of latitude in the Indian Ocean. Using Bayesian meta-analysis we show that changes in the size structure, diversity and trophic composition of the reef fish community have followed coral declines. Although the ocean scale integrity of these coral reef ecosystems has been lost, it is positive to see the effects are spatially variable at multiple scales, with impacts and vulnerability affected by geography but not management regime. Existing no-take marine protected areas still support high biomass of fish, however they had no positive affect on the ecosystem response to large-scale disturbance. This suggests a need for future conservation and management efforts to identify and protect regional refugia, which should be integrated into existing management frameworks and combined with policies to improve system-wide resilience to climate variation and change.

## Introduction

Coral reefs are one of the ecosystems most threatened by climate variability and change [1]–[3]. Reef corals, the building blocks of carbonate reefs, have a restricted thermal tolerance, resulting in ‘bleaching’ events (loss of symbiotic algae) when sea surface temperatures rise above a given threshold [4]. This has contributed to widespread loss of live coral cover [5]–[8], the restructuring of benthic community composition [9] and has resulted in dire predictions for the future persistence of coral-dominated ecosystems within decadal time scales [10], [11]. There is now a need to understand resultant large-scale implications for other components of the ecosystem, which, to date, have received limited attention or been the focus of local studies [12]–[14]. Assessing ecosystem trends and patterns at regional scales is necessary if informed management choices are to be made that will mitigate the effects of large-scale climate disturbance. Importantly, there is a need to test key paradigms, such as the ability of no-take areas (NTAs) to enhance recovery from climate change impacts [2], and the potential for herbivorous fish to increase in abundance following coral mortality and functionally compensate for increased algal coverage [15].

At large scales, remote pristine areas may have a greater capacity to absorb climate impacts and maintain a coral dominated and diverse ecosystem [16]. However, most coral reef NTAs are small and embedded in heavily fished and degraded environments [8], [17]. Assessing the importance of local management for conserving coral reefs in the context of global change has been identified as a key research challenge for coral reef scientists [18]. Although there are expectations that NTAs will promote resilience and faster recovery from climate disturbance [19], site-specific studies suggest this may not be the case [12], [20], [21], and the effectiveness of such management needs to be assessed across regional spatial scales.

Grazing by herbivores, by creating space for invertebrate larval settlement, is thought to be key to maintaining reefs in a coral dominated state [22], [8], [23]. However, it is increasingly evident that the majority of herbivorous fish in the Indo-Pacific will crop turf algae, but feed less on or avoid erect macroalgae once it has developed [24], [25]. Following large-scale disturbances that open up large amounts of space on reefs, such as mass coral bleaching, herbivores may become swamped by the biomass of the new algal resource [26] and reefs can progress on a trajectory to macroalgal dominance [27]. It is therefore important to assess whether herbivorous reef fish increase in abundance following large-scale coral loss and thus have the ability to prevent reefs from becoming dominated by erect macroalgae.

Coral mortality through climate induced bleaching was particularly severe in the Indian Ocean in 1998, with ∼45% of coral cover lost across the region [28], although the effects were spatially variable [7], [9]. We assess the longer-term effects of this event in fished areas and NTAs across 7 countries, 66 sites and 26 degrees of latitude. Specifically, we conducted a targeted research program whereby the original investigators who collected comprehensive benthic and fish assemblage data from Maldives, Chagos, Seychelles, Kenya, Tanzania, Mauritius, and Réunion in the mid-1990s repeated their surveys post-bleaching, in 2005. We use continuous model Bayesian meta-analysis to quantify effects of changes in live coral cover and physical complexity of reefs on the diversity, size structure, trophic structure and abundance of reef fish. The Bayesian approach not only structures the inherent uncertainty in monitoring data from multiple sources, but also allows belief statements to be made regarding future change [29]. With ever more frequent bleaching events predicted [11], quantitative predictions regarding how fish will respond to future declines in coral cover over large spatial scales are needed to guide regional conservation planning, adaptation and mitigation strategies.

## Results

**Figure 1:**
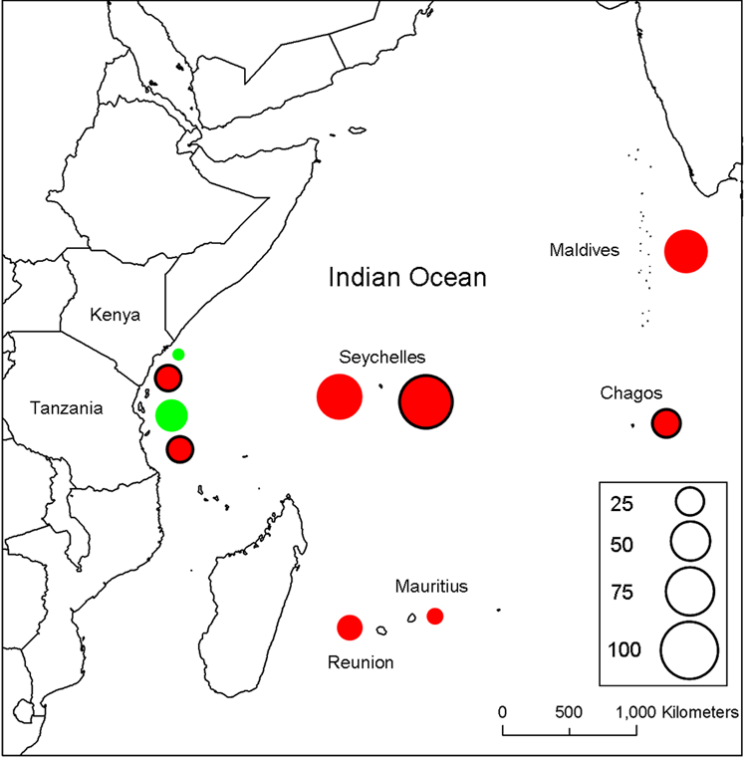
Change in coral cover at sites across the western Indian Ocean. Green and red symbols represent increases and decreases in coral cover respectively. Symbols with solid borders are sites in NTAs; Seychelles data include two NTAs, Kenya includes four, Tanzania two and the Chagos archipelago is a de-facto NTA. Data represent 66 sites across the region. Numbers in key (size of bubble) are percent changes between mid 1990s and 2005. Map produced using ESRI data and ArcGIS 9.

**Figure 2:**
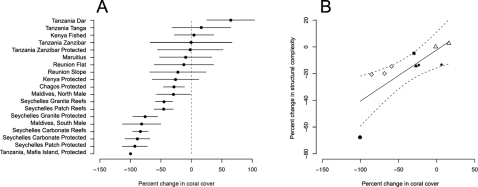
Change in coral cover and reef structural complexity. (*A*) Change in live coral cover at meaningful biogeographical aggregations and by management strategy. Three habitat types in Seychelles each replicated in the two NTAs. Kenyan protected represents four NTAs. Bootstrapped 95% confidence intervals indicate whether mean change departs significantly from zero. Locations ordered by magnitude of coral decline. (*B*) Correlation between change in live coral cover and change in structural complexity across the region. • Mafia Island, ◊ Seychelles, ▴ Chagos, ▪ Maldives, ♦ Kenya, ▵ Tanzania.

Change in hard coral cover across the region between the mid 1990s and 2005 varied geographically (Figure 1). The changes reported here represent the combined effects of coral loss in 1998 and any subsequent recovery to 2005. The greatest declines were apparent through the low latitude island states of Maldives, Chagos, and Seychelles. Kenyan and Tanzanian nationally protected sites experienced moderate declines, while Mauritius and Réunion sustained the smallest declines, and coral cover increased in Kenyan and Tanzanian fished sites (Figure 1). Assessing change in coral cover at relevant scales, that consider location, management and habitat type, indicates that 10 of our 19 study locations exhibit declines that depart significantly from zero (Figure 2A). The study incorporated nine no-take areas (NTAs) across four countries (two in Seychelles, four in Kenya, two in Tanzania and the long-term de-facto protection of reefs of the Chagos archipelago [30]). A greater proportion of NTAs (71%) than fished (42%) locations showed significant declines in coral cover over the study period. Based on bootstrapped 95% confidence limits, there was no evidence to suggest the percent change in coral cover differed between NTAs and fished areas, and in some cases declines were significantly greater in NTAs (Figure 2A). Importantly, the NTAs had greater starting coral covers than adjacent fished areas, which, as NTAs and fished areas declined to similar final covers (Table 1)(with the exception of some of the less impacted Tanzanian sites), meant the NTAs had further to fall.

**Table 1:** Mean coral cover before (mid-1990s) and after (2005) the 1998 bleaching event across the Indian Ocean.

Location	% Coral Cover mid-1990s	±SE	% Coral Cover 2005	±SE
Maldives, North Male (3)	15.5	7.5	10.9	3.2
Maldives, South Male (2)	43.9	3.6	8.0	1.2
Chagos Protected (9)	31.2	4.0	22.8	2.9
Seychelles Carbonate Reefs (5)	34.6	2.7	5.6	3.1
Seychelles Carbonate Protected (2)	44.9	4.8	5.1	4.5
Seychelles Granite Reefs (5)	14.8	2.0	8.2	2.3
Seychelles Granite Protected (2)	30.9	7.6	7.5	6.4
Seychelles Patch Reefs (5)	20.0	1.5	10.9	5.1
Seychelles Patch Protected (2)	46.4	7.8	3.6	3.0
Kenya Fished (4)	18.9	5.2	20.0	4.0
Kenya Protected (4)	34.8	4.5	26.8	8.1
Tanzania Dar (4)	42.6	11.9	70.0	3.2
Tanzania Tanga (4)	23.9	7.5	27.8	6.8
Tanzania Zanzibar (2)	48.5	3.8	48.3	3.3
Tanzania Zanzibar Protected (2)	62.7	11.1	61.5	2.4
Tanzania, Mafia Island, Protected (2)	33.0	N/A	0.1	N/A
Reunion Flat (2)	42.5	24.3	37.0	10.9
Reunion Slope (2)	42.0	5.0	28.4	4.5
Mauritius (5)	45.3	9.5	41.1	6.7

Sites aggregated at representative geographic scales that consider location, management and habitat type. Three habitat types in Seychelles each replicated in the two NTAs. Kenyan protected represents four NTAs. Number of sites per location given in brackets. Note, Tanzania, Mafia Island, received no-take status in 2000.

It is clear that the impacts of the 1998 bleaching event were highly variable across the region, and provide a continuum against which to test secondary consequences, such as the effects of coral loss on fish assemblages. Recent developments in assessing the effects of coral disturbance on fish have highlighted the importance of eroding structural complexity in driving responses [13], [31], which, as erosion of coral structures can take 5–10 years, explains the much smaller impacts on fish shortly after coral mortality [15]. Structural complexity was quantified at 50 of our 66 sites. Importantly, there was a strong correlation between loss in coral cover and loss in structural complexity across the region (*r* = 0.77, P<0.001, Figure 2B). The strong collinearity in the two measures precludes independent assessment of variables, and therefore the effects of changing coral cover on fish identified in the Bayesian meta-analyses are likely to result from a combination of loss in coral cover and structural complexity.

**Figure 3:**
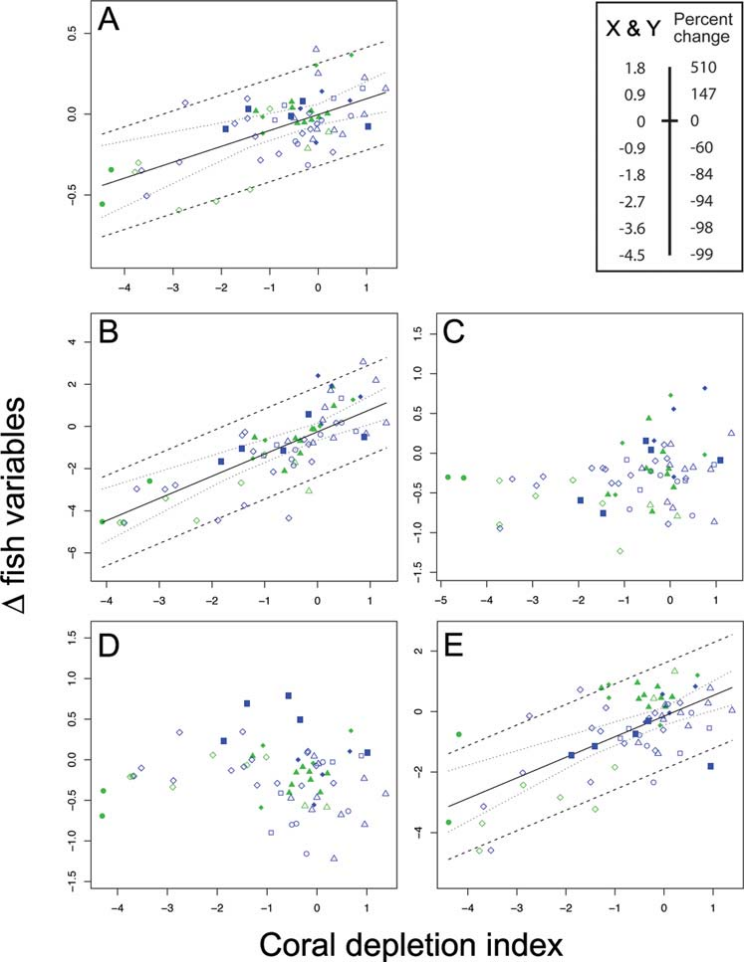
Change in fish groups in response to coral decline. Continuous model Bayesian meta-analysis of relationships between decline in coral cover and change in (*A*) species richness of fish assemblages, and (*B*) abundance of obligate corallivores, (*C*) herbivores, (*D*) mixed diet feeders, (*E*) planktivores. Scale as converted to percent change indicated in top right panel. Linear trend lines only presented where significant model fits were recorded. Green symbols indicate sites in NTAs, blue symbols indicate sites in fished areas. Inner dashed line represents 95% credible interval on the regression and outer dashed line represents the 95% prediction interval. • Mafia Island, ◊ Seychelles, ▴ Chagos, ▪ Maldives, ♦ Kenya, ▵ Tanzania, □ Réunion, ○ Mauritius. Movement of points along the x-axis among panels reflects model-structured uncertainty present among studies.

Coral loss predicted declines in reef-fish species richness, and abundance of obligate corallivores, planktivores and fishes <20 cm throughout the western Indian Ocean (Table 2). We tested five possible trajectory descriptors in each case, but only found evidence for linear fits between coral decline and change in groupings of the fish community. Trends in species richness were significant, but weak, and largely driven by the Seychelles and Mafia Island (Figure 3A). There was substantial evidence for a 1∶1 relationship between changes in obligate corallivore abundance and percent coral cover (Figure 3B). From these results we estimate, given any future 50% decline in coral cover, there is a 76% probability of equivalent declines in obligate corallivores at any given site in the western Indian Ocean. The relationship between change in diurnal planktivore abundance and coral cover was relatively strong; given a future 50% decline in coral cover, we estimate a 68% probability of observing declines in planktivore abundance (Figure 3E). We found no relationship between a loss of coral and change in abundance of herbivore and mixed diet feeder groups (Figure 3C,D).

**Table 2:** Continuous model Bayesian meta-analysis parameter estimates from the best-fitting models (see Table 3) for reef fish metrics in the western Indian Ocean.

Metric	Model	*βˆ*_0_ (intercept)	*βˆ* *_coral_* (slope)	*βˆ* *_protected_* (intercept)	*βˆ* *_protected_* (slope)
Species richness	M_c_	0.00 (0.03) [−0.07, 0.06]	0.10 (0.02) [0.06, 0.14]	-	-
Obligate corallivores	M_c_	−0.26 (0.21) [−0.66, 0.16]	1.05 (0.14) [0.77, 1.30]	-	-
Herbivores	M_0_	−0.28 (0.08) [−0.45, −0.12]	-	-	-
Mixed-diet feeders	M_0_	−0.18 (0.06) [−0.28, 0.08]	-	-	-
Planktivores	M_cp_	−0.42 (0.16) [−0.74, −0.10]	0.52 (0.16) [0.28, 0.77]	1.02 (0.35) [0.35, 1.69]	0.61 (0.24) [0.10, 1.07]
Planktivores	M_c_	−0.15 (0.15) [−0.44, 0.14]	0.68 (0.10) [0.48, 0.87]	-	-
<20 cm	M_c_	−0.17 (0.09) [−0.35, 0.00]	0.37 (0.06) [0.28, 0.49]	-	-
21–40 cm	M_0_	−0.21 (0.09) [−0.40, −0.05]	-	-	-
41–60 cm	M_0_	−0.59 (0.17) [−0.91, −0.26]	-	-	-
>61 cm	M_0_	−0.37 (0.21) [−0.77, 0.04]	-	-	-
<20 cm obligate corallivores	M_c_	−0.39 (0.19) [−0.77, −0.00]	0.94 (0.13) [0.69, 1.19]	-	-
<20 cm herbivores	M_c_	−0.19 (0.16) [−0.51, 0.13]	0.50 (0.10) [0.29, 0.71]	-	-
<20 cm herbivores	M_cp_	−0.28 (0.18) [−0.65, 0.08]	0.24 (0.14) [−0.05, 0.53]	0.37 (0.35) [−0.33, 1.06]	0.53 (0.21) [0.12, 0.95]
<20 cm mixed diet feeders	M_0_	−0.46 (0.06) [−0.58, −0.34]	-	-	-
<20 cm planktivores	M_c_	−0.10 (0.16) [−0.43, 0.23]	0.57 (0.110 [0.35, 0.78]	-	-

Estimates for groups with equivalent model fits are provided for both models. Values in parentheses are standard deviations; values in square brackets are 95% credible intervals.

**Figure 4:**
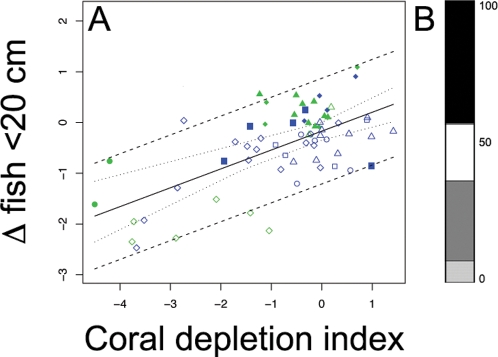
Change in small bodied fish in response to coral decline. (*A*) Continuous model Bayesian meta-analysis of relationship between decline in coral cover and change in fish <20 cm maximum attainable size. Green symbols indicate sites in NTAs, blue symbols indicate sites in fished areas. Inner dashed line represents 95% credible interval on the regression and outer dashed line represents the 95% prediction interval. • Mafia Island, ◊ Seychelles, ▴ Chagos, ▪ Maldives, ♦ Kenya, ▵ Tanzania, □ Réunion, ○ Mauritius. (*B*) Percent contribution of five trophic groups to the starting (mid-1990's) abundance of fish <20 cm maximum attainable body length across the region. Black = planktivores, dark grey = piscivores (barely present on plot; 0.05%), white = Mixed diet feeders, grey = herbivores, light grey = obligate corallivores.

When species were grouped by their maximum attainable size, a clear trend was apparent for species <20 cm total length, but no relationship was observed for 21–40 cm, 41–60 cm or >60 cm groupings (Figure 4A; Table 2). Given a future 50% decline in coral cover, we estimate a 52% probability of observing declines in the abundance of fish species with maximum body lengths <20 cm. Within this size class, planktivores make up a considerable portion of the abundance (44%), and herbivores and mixed diet feeders also contribute substantially (28% and 20% respectively), but corallivores have limited input (8%) (Figure 4B). Separate analyses of trophic groups within the <20 cm size category highlights that, along with obligate corallivores and planktivores, there was also evidence of declines in herbivores (Table 2).

We only found weak evidence for differences between NTAs and fished areas for change in diurnal planktivore abundance and small-bodied herbivore abundance (<20 cm) (Table 3). In both cases the negative relationship between fish abundance and coral decline was greater for the NTAs, however there was equal support for model M_c_ with no differences between types of management (Table 3). Importantly, irrespective of body size and trophic categorization, NTAs provided no clear benefits for any of the fish groups in terms of their change in response to coral decline.

**Table 3:** Model-selection results for continuous model Bayesian meta-analysis in the western Indian Ocean using the Bayesian Information Criterion (BIC).

Metric	M_0_	M_c_	M_cp_
Species richness	17.53	**1.64**	6.35
Obligate corallivores	282.90	**248.38**	256.38
Herbivores	**145.83**	149.61	157.35
Mixed-diet feeders	**83.64**	87.40	91.90
Planktivores	237.66	**205.27**	**204.30**
<20 cm	165.25	**138.72**	140.60
21–40 cm	**146.91**	150.00	158.17
41–60 cm	**231.69**	**231.35**	235.95
>61 cm	**261.85**	266.02	272.32
<20 cm obligate corallivores	275.96	**238.91**	246.80
<20 cm herbivores	230.91	**214.86**	**216.53**
<20 cm mixed diet feeders	**94.93**	99.11	106.16
<20 cm planktivores	237.76	**218.23**	222.13

Models include a null model of no relationship (M_0_), a model including a relationship between changes in coral cover and reef fish metrics (M_c_), and a fish-coral model that allows for differences between protected and unprotected sites (M_cp_). Models highlighted in bold have the greatest support, given the data; models with BIC differences of <2 are considered to have equal support.

## Discussion

We have identified spatially variable declines in coral cover, reef structural complexity, fish species richness and the abundance of various feeding and size groups of reef fish across the Indian Ocean following the 1998 bleaching event. These changes are substantial for some groups, and indicate little insurance offered by current small-scale NTA management across the region. The spatial patterns present in our data provide important information for future conservation planning and generic lessons for managing whole coral reef ecosystems in a changing climate.

There was little difference in the decline of coral cover between NTAs and fished areas across the Indian Ocean, with some evidence for greater declines within NTAs. This result is likely due to NTAs often being sited in areas where the cover of *Acropora* and other thermally-sensitive and branching coral species is high [20], or may be because fishing gears reduce cover of these coral species in fished areas. Our analysis also indicated little difference between NTAs and fished areas for those fish groups that declined in response to coral loss. The only indication of a differential response was the greater decline in NTAs for planktivores and small bodied herbivores. Large, remote and pristine areas seem to be resilient to a wide range of disturbances [16], which has led to calls to assess the effectiveness of NTAs in conserving coral reefs through climate disturbance [18]. One clear difference to these remote areas is that NTAs on reefs are typically small and surrounded by much larger areas that are modified by exploitation [8], [17]. As we do not have repeat temporal data since the initial coral loss in 1998, we can not explicitly infer recovery rates from our data, however the NTAs we studied show no evidence of being more resistant to declines in coral and fish groups following coral bleaching and it seems likely that, over this time scale, recovery rates are no different between NTAs and fished areas, as has been shown for some of the NTAs where temporal data were available [21].

We detected declines in fish species richness across the western Indian Ocean in response to loss of live coral cover. Although only a small proportion of species are heavily coral dependent, most species are reliant on the reef matrix at some stage in their life history, and change in species richness was likely due to loss in the physical structure of the reef, rather than live coral [13]–[15], [31]. The variability in loss of structural complexity may explain why the trend for species richness was not stronger, with locations such as Chagos, where recovery of coral has been rapid, potentially retaining structural complexity in the interim. Although loss of structural complexity was the most likely driver of the region-wide decline in species richness, some studies have highlighted that live coral can be an important settlement cue for larval fish [12], [32] and the nature of this relationship is an important area for future research.

Although previous studies have identified obligate corallivores as a functional group vulnerable to declines in coral cover [14], [15], this is the first study to demonstrate declines over such a large spatial scale. We have also identified a 1∶1 linear relationship between coral loss and obligate corallivore decline, suggesting their survival on the reef is tightly linked to coral cover and changes in obligate corallivore abundance should be easy to predict based on changes to benthic cover. The diurnal planktivores in the study were largely small-bodied species from the damselfish family (>90% contribution to group) that are often closely associated with the reef matrix [33], [34]. Their decline is most likely due to predation vulnerability, linked to loss of coral and structural collapse [13], [31]. Planktivores and corallivores showed the strongest relationships of all groups to declining coral cover and are likely to be the groups most threatened from the predicted ongoing decline in global reef health [14], [15].

Although herbivores are hypothesized to increase in abundance following coral decline due to a greater availability of algal resources, previous studies have reported high variation in this relationship and have often been conducted shortly after disturbances, limiting their ability to detect demographic changes [15]. Here we tested this hypothesis across large spatial and temporal scales where the assemblage had a moderate time to respond. Herbivores are thought to be a key functional group, responsible for the resilience of reef systems by controlling algal growth [8], [23], [35] and ultimately allowing settlement of new coral recruits [36]. However, our data show that the proliferation of algae that follows extensive coral mortality [12], [13], [37], [38] was unlikely to be controlled by a corresponding increase in herbivorous fish abundance. Changes to size structure and biomass of herbivore stocks cannot be ruled out and may initially encourage increased consumption and control of algae. However, studies from Seychelles suggest such changes may be indicative of future declines in herbivore abundance and biomass due to a loss of refuge from predators, leading to reduced recruitment to adult size classes [20].

The mixed diet feeding group also showed no response to declining coral cover. This group of fish includes species from families such as Lethrinidae, Mullidae, Lutjanidae, and Labridae, many of which are habitat generalists, foraging and recruiting to non-coral reef habitats such as seagrass [39]. Species in these groups also tend to forage over fairly large spatial scales, indicating a lack of reliance on specific habitat types. Due to this decoupling of reliance on reef habitat and the potential benefits they may glean from increased food resources, this may be the group that will be sustained in the long term, although a large amount of variation can be expected at the species level [15], leading to changes in community composition.

Small-bodied fish are known to be more reliant on the reef matrix, inhabit narrower niches, and be more vulnerable to predation [33], [34]. Our analyses highlight the vulnerability of small-bodied species to coral and structural complexity loss. Within this size category, obligate corallivore and planktivore groups showed strong declines. Interestingly, there was also a reduction in abundance of small-bodied herbivores. Although herbivore abundance may not be declining overall (Figure 3C), the reduction of these small-bodied species is of concern as they perform important functional roles on coral reefs [40]. Small mixed diet feeders again showed no trend, demonstrating the resistance of species with generalist life history traits to coral loss.

There are some obvious limitations in our data, such as the timeframe between surveys and the influence of any change in management / fishing pressure. In most cases management and fishing pressure have not changed greatly over the ten years studied. The one main exception is Mombasa Marine National Park, Kenya, where species richness and fish density have increased owing to management action [17]. Although such effects may have a slight influence on the results, the relationship between reef fish and change in coral cover (and its association with loss in physical structure) is a strong signal within the regional data and is consistent with current ecological understanding of disturbance effects on coral reefs [14], [15]. A potential problem when conducting meta-analyses is publication bias, whereby data sets are not located or included in the analyses [41]. This is not a problem in the current study as we conducted a targeted research program where all comprehensive studies from the mid 1990's were repeated as part of the study itself. Finally, the study design does not consider the impact of disturbances after the 1998 coral bleaching event. However, the December 2004 tsunami is thought to have had negligible effects on coral reefs in the western Indian Ocean [42]. Furthermore, any other ensuing disturbances are just as likely to have influenced NTAs as fished areas and reflect increasing disturbance frequencies occurring on coral reefs globally [2], [8].

Our analyses highlight great geographic variation in the impact of coral bleaching across the region, with the Seychelles suffering the greatest in terms of coral loss and associated effects on fish, and the Mascarene Islands (Réunion and Mauritius) suffering the least. These trends could be due to several factors: 1) Prevailing currents and variation in temperatures have been identified as key determinants of coral mortality in the region, likely reducing mortality in the Mascarene Islands in particular [11], [43]. 2) Well connected reef systems are expected to contain the pockets of refugia required for landscape-scale recovery [44]. This is evident when comparing recovery of the well connected mainland reefs of Kenya and Tanzania and the geographically extensive Chagos and Maldives to the geographically small and isolated inner Seychelles. 3) The inner Seychelles is a shallow continental shelf basin, with most fringing reefs extending to only 7–9 m depth. This ‘bathtub effect’ likely led to extensive mortality in 1998 and precluded any depth refuge below which corals could survive. Where live coral extends to 40–50 m depth, such as in the atolls of Chagos or the islands of Réunion and Mauritius, a depth refuge of broodstock may encourage faster recovery of corals at shallower depths [45]. 4) Finally, the atolls surveyed in Chagos are uninhabited and off limits to reef fishing. The lack of multiple anthropogenic stresses that most other reef systems endure may have helped promote recovery from the disturbance [16], [18].

The 1998 bleaching event had, and is still having, extensive effects across the western Indian Ocean. Although ocean-scale coral reef integrity has been lost, it is positive to see that effects were spatially variable and that in some locations the indirect effects on fish assemblages and likely implications for human society have been small. Geography seems to be a key determinant in the ability of reefs to absorb and recover from such large-scale disturbances and this should be considered for other regions likely to suffer similar large-scale disturbances in the future. Although there was no evidence that existing NTAs are promoting recovery of coral, these NTAs are still supporting a greater biomass of fishery stocks [17], [20], indicating long-term fisheries management should not be compromised. There is, however, a need for new NTAs, incorporated into existing networks that protect source reefs resilient to large-scale disturbance, and areas likely to retain their physical structure. This will help sustain the upstream spawning stocks of corals and specialised fish species required for landscape-scale recovery. Such management is likely to be unsuccessful in isolation, and improved management of entire reef systems, reducing the stresses and pressures to areas outside NTAs will be necessary to maximise the capacity for systems to recover from large scale and ongoing disturbance.

## Materials and Methods

We identified all field studies that had comprehensively surveyed reef fish assemblages and associated benthic composition and structure from the western Indian Ocean region from 1990 to before the 1998 coral bleaching event (majority 1994–95). This resulted in eight separate large-scale studies (across seven countries). Original investigators returned to their study locations in 2005 to repeat the surveys, using field protocols identical to those used in the original surveys. The protocols were standardised within, rather than among study locations as it is more robust to quantify effect sizes in this way and then standardise when comparing among studies. Where the original investigator could not return, an experienced surveyor from the team repeated the work. An associated field study workshop for the project, which involved many of the researchers from the region, found experienced observer bias to be a very small component of the variation in fish counts [46]. All reef surveys were conducted on the reef flat or shallow reef slope. The abundance of all diurnally active, non-cryptic, reef-associated fish was assessed during each survey, however methods varied among study locations from point counts of differing dimensions to belt transects of differing dimensions. Replication also varied from 3 to 16. This resulted in a survey area per site of ∼200 m^2^ to ∼2500 m^2^. Benthic quantification also varied in spatial scale and from visual estimates to line intercept transects, but the results are expected to be comparable [47]. Estimates of change in live coral cover were calculated and plotted on a map by country and management strategy and at a more aggregated level with 95% confidence limits. Measures of structural complexity also varied and included visual assessments of reef topography, the linear versus contour method and measures of reef height. However these measures were found to be strongly correlated [47] and these correlation coefficients were used to standardise them to a common scale. The relationship between percent change in coral cover and percent change in structural complexity was assessed by correlation analysis. The presence of variation in field methods is routine in meta-analytical studies, and thus the choice of effect size calculation and variance weighting is integral to the comparability of study results [48].

### Effect size

Meta-analysis frequently employs unitless effect size metrics to standardize the information present among accumulated studies. The potential to observe changes in a before and after comparison can be greatly influenced by initial values at a given location; sites with larger initial values have a greater scope to reveal change than those with low values [48]. To achieve a comparable metric at all locations and to account for initial cover / values, we calculated effect sizes as the percent change between the mid 1990s and 2005 [49];
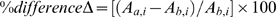
(1)where *A_b_* and *A_a_* were mean values at sites in the mid 1990's and 2005 respectively. We did not account for study duration [48] as we made the informed assumption that the greatest changes occurred in 1998 and our measures in the mid-1990's are an appropriate estimate of pre-bleaching conditions. Furthermore as sampling date was standardised for post-1998 surveys, any incorporation of duration could unduly bias effect sizes based on pre-disturbance study dates. Finally, we are estimating a magnitude of change, rather than a rate of change, which would require a different effect size metric [48]. We calculated individual effect sizes for change in coral cover, structural complexity, fish species richness, and fish density in four functional groups for which data were available at the majority of sites (obligate corallivores, herbivores, planktivores, and mixed-diet fishes assigned using regional fish identification guides, published literature and http://www.fishbase.org), for four size classes of fish species (maximum attainable size <20 cm, 21–40 cm, 41–60 cm, and >60 cm) and for the same four functional groups listed above within the <20 cm maximum attainable size category. Herbivores include all those species that feed on algae and or detrital aggregates from the epilithic algal matrix. Because percent-change losses have a strongly right-tailed distribution, i.e. a maximum potential decline of 100%, but a potentially limitless increase, we transformed all of the Δ*^T^* values to be balanced around zero following Kaiser et al. [49]:
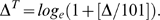
(2)This transformation prevents overestimates of increases and underestimates of declines, where a maximum potential decline has a value of −4.6 and a maximum increase +4.6. The transformation approximately normalises the error distribution and stabilises its variance [49]. Raw data were available for many of the original studies, allowing us to estimate average effect-sizes at some locations. Because data were collected from the same sites but not the same transects, we estimated effect-size means and variances at these sites using non-parametric bootstrapping of the before and after observations (R = 9999) [50] with (1) and (2), by randomly matching before-after pairs at each iteration. This generated sample means and expected variance ranges for many, but not all, of the study locations.

### Bayesian meta-analysis

We evaluated evidence for a regional relationship between reef fish and coral cover using an area-variance weighting scheme implemented in a Bayesian meta-analysis framework. The use of area surveyed as a weighting scheme in coral reef meta-analyses has become widespread because actual variance will depend on individual measurement size and replication [48]. The Bayesian approach allowed us to model the hierarchical structure of the data, estimate the magnitude of regional-scale effects, and to specify a level of uncertainty about individual study estimates. By sharing information among studies, this approach maximized the strength of inferences made across the entire range of meta-data used, allowing us to make probability statements about the likelihood of reef fish declines given potential future changes in coral conditions. Although we tested five different ecologically meaningful response trajectories (asymptotic, quadratic, logistic, linear and exponential), we found no model-based evidence for non-linear responses based on Bayesian Information Criterion (BIC) scores among candidate models. We therefore quantified the regional fish community response between the mid 1990s and 2005 using a null model (intercept-only; M_0_) and exchangeable linear model (M_c_) of coral effect size *β_coral_*,
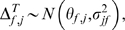
(3)

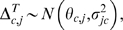
(4)

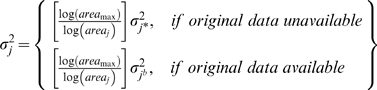
(5)

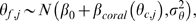
(6)where 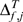 is the study point estimate of the fish effect size θ*_f_*_,*j*_ from the *j^th^* study; 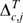 is the study point estimate of coral effect size *θ_c_*_,*j*_ in the *j^th^* study; 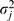 is the study fish or coral variance that is assumed known; 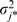 is the maximum of the known (bootstrap estimated) site-level variances for fish or coral among the studies used; *area_max_* is the maximum reef area surveyed; 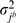 is the bootstrap-estimated site-level variance for sites where raw data was available; and 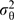 is the estimated regional variance. The area-weighted 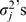 were likely to be conservative because they were scaled down from the largest known study variance, expressing an equal or greater level of uncertainty than any of the known sample variances, thus weighting the variance based on the area of reef surveyed.

This continuous meta-analysis model was fully-specified by non-informative prior distributions for the estimated parameters,
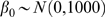
(7)

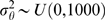
(8)

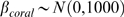
(9)In addition to the coral effects model, we included a NTA model to estimate the effects of fishery protection on changes in coral and fish metrics. This protection model (M_cp_) included a modification of equation (6) to include a dummy variable (*status*) that allowed the slopes (*β_protection_*) and intercepts (*β_prot _*_0_) of the coral relationship to vary between NTAs and fished areas:
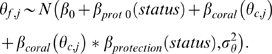
(10)Priors for all slopes and intercepts were as specified by equation (9). We implemented both regional models using the PyMC Markov-Chain Monte Carlo (MCMC) toolkit for the Python programming language. Meta-analytical models were run for 20 000 iterations with a 10 000 iteration burn-in period. We evaluated model convergence using Geweke's method [50]. Model goodness-of-fit (GOF) was assessed using the deviance simulation methods in PyMC, where ideal models yield GOF values near 0.5, providing evidence of equivalence between simulated and observed deviance [51]. Our Bayesian meta-analyses had GOF scores between 0.46 and 0.50 for all fish metrics, confirming good model fits for estimating effect-size relationships, and model convergence was deemed adequate in every instance [51]. Site-level posterior distributions shrunk towards the regional mean, where the extreme high- and low-value effect sizes had a reduced effect on the overall estimates. Relative evidence for each model was evaluated using the Bayesian Information Criterion (BIC) [52] and the uncertainty surrounding each posterior parameter estimate.

From the area of highest posterior density in the posterior distribution of each model parameter we obtained Bayesian credible intervals (CI) that defined a 95% probability of a given parameter lying within the CI range. During each MCMC simulation we also sampled from the full conditional of the model and data to construct predictive intervals (PI) that defined a 95% probability of future observations being within the PI range. The PI interval values allowed us to make probability statements about the response of fish assemblage groups to future coral depletion.
